# Chanarin-Dorfman Syndrome (CDS): A Rare Lipid Metabolism Disorder

**DOI:** 10.7759/cureus.43889

**Published:** 2023-08-21

**Authors:** Nisarg P Mangukiya, Safa Kaleem, D Ragasri Meghana, Lyluma Ishfaq, Gunjan Kochhar, Bejoi Mathew, Shivani Pulekar, Aashka C Lainingwala, Mihirkumar P Parmar, Vishal Venugopal

**Affiliations:** 1 Internal Medicine, Gujarat Medical Education & Research Society Medical College, Vadnagar, IND; 2 Internal Medicine, Shadan Institute of Medical Sciences, Hyderabad, IND; 3 Internal Medicine, Kakatiya Medical College, Warangal, IND; 4 Internal Medicine, Directorate of Health Services Kashmir, Srinagar, IND; 5 Internal Medicine, Punjab Institute of Medical Sciences, Jalandhar, IND; 6 Internal Medicine, Sri Devaraj Urs Medical College, Kolar, IND; 7 Internal Medicine, Davao Medical School Foundation, Davao, PHL; 8 Internal Medicine, Bhaarath Medical College & Hospital, Chennai, IND

**Keywords:** dorfman, ascites, congenital, rare skin disease, chanarin

## Abstract

Chanarin-Dorfman syndrome (CDS) is a rare medical condition that is inherited in an autosomal recessive pattern. In CDS, a comparative gene identification-58 gene mutation causes the accumulation of triglycerides in neutrophils, which can be observed as vacuoles on a peripheral smear. CDS patients present with a characteristic dermatological finding, ichthyosis, which is a non-bullous white scaling of the skin. Here, we describe a case report of a one-year-old boy who presented to the pediatric outpatient department (OPD) with chief complaints of peeling of the skin and ballooning of the abdomen since birth. Our patient had achieved all the developmental milestones pertaining to his age. Genetic testing was positive for heterozygous alleles in both parents.

## Introduction

Chanarin-Dorfman syndrome (CDS) is an uncommon autosomal recessive disorder that affects multiple organ systems [1]. This hereditary condition is distinguished by an excessive accumulation of neutral lipid, triacylglycerol, in many tissues and organs throughout the body, contributing to an extensive spectrum of symptoms that are inherently due to mutations in an abhydrolase domain-containing gene 5, otherwise known as the comparative gene identification-58 (CGI-58) gene, positioned on chromosome 3 [2-3]. The disease is named after Dorfman and Chanarin, who first described it in 1974 and 1975, respectively. It is characterized by lipid accumulation in peripheral blood leukocytes, bone marrow granulocyte precursors, liver cells, and many other cells in the body. However, it was Jordan who first discovered lipid vacuoles in the cytoplasm of leukocytes from a peripheral blood smear of two brothers suffering from progressive muscular dystrophy in 1953 [3]. ABDH5 is a multimeric protein that activates intracellular lipases such as adipose triglyceride lipase (ATGL), which plays a significant role in lipolysis. Simulations using machine learning-based homology modeling methods have suggested that mutations in genes E41, R116, and G328 disturb hydrogen bonding networks and suppress ATGL activation [4]. CDS has also been described as a “neutral lipid storage disease (NLSD) with ichthyosis” owing to the neutral lipids that are deposited in a variety of organs, including the skin, muscle, liver, central nervous system, and granulocytes [5]. Typically, CDS patients present with ichthyosis, hepatomegaly, cirrhosis, cardiomyopathy, splenomegaly, and myopathy. Less common features, such as cataracts, keratopathy, hearing loss, and mental retardation, have also been observed [1], and management is primarily symptomatic. Recommendations include a diet low in long-chain fatty acids and saturated fats. Emollients help with skin manifestations. Patients who do not have impaired liver functions can also opt for acitretin [6].

Since its discovery, fewer than 120 cases of CDS have been reported in the literature, particularly in Mediterranean and Middle Eastern countries [7-9], especially Turkey, with a few cases also reported in China, making it an extremely rare disorder. Despite its rarity, CDS poses a substantial challenge to clinicians due to its broad spectrum of symptoms. Therefore, this case report aims to discuss the clinical presentation, diagnosis, and management of CDS in our facility in India.

## Case presentation

A one-year-old boy presented to the pediatric outpatient department (OPD) with complaints of skin peeling and abdominal enlargement, both of which were progressively increasing. Since birth, the patient has had peeling skin all over his body but particularly on his face. His complexion had a light-brown hue. In addition, the patient had a white discoloration of the lens in the ocular. The patient had a history of phototherapy-cured neonatal jaundice at the age of one month. The patient had no analogous family history. The mother and father of the patient had a non-consanguineous marriage. His mother suffered from gestational diabetes and was treated with insulin during her pregnancy. After delivery, the mother’s blood sugar level was within normal limits, two tetanus toxoid doses were administered, and she consumed calcium and iron-folic acid supplements. The patient's birth history was a vaginal delivery at full term with no immediate complications. The patient began to cry immediately after birth. He was fully immunized when he was one year old. The patient reached all development milestones without any delay. The patient was exclusively breastfed for six months and started weaning around this time. 

**Figure 1:**
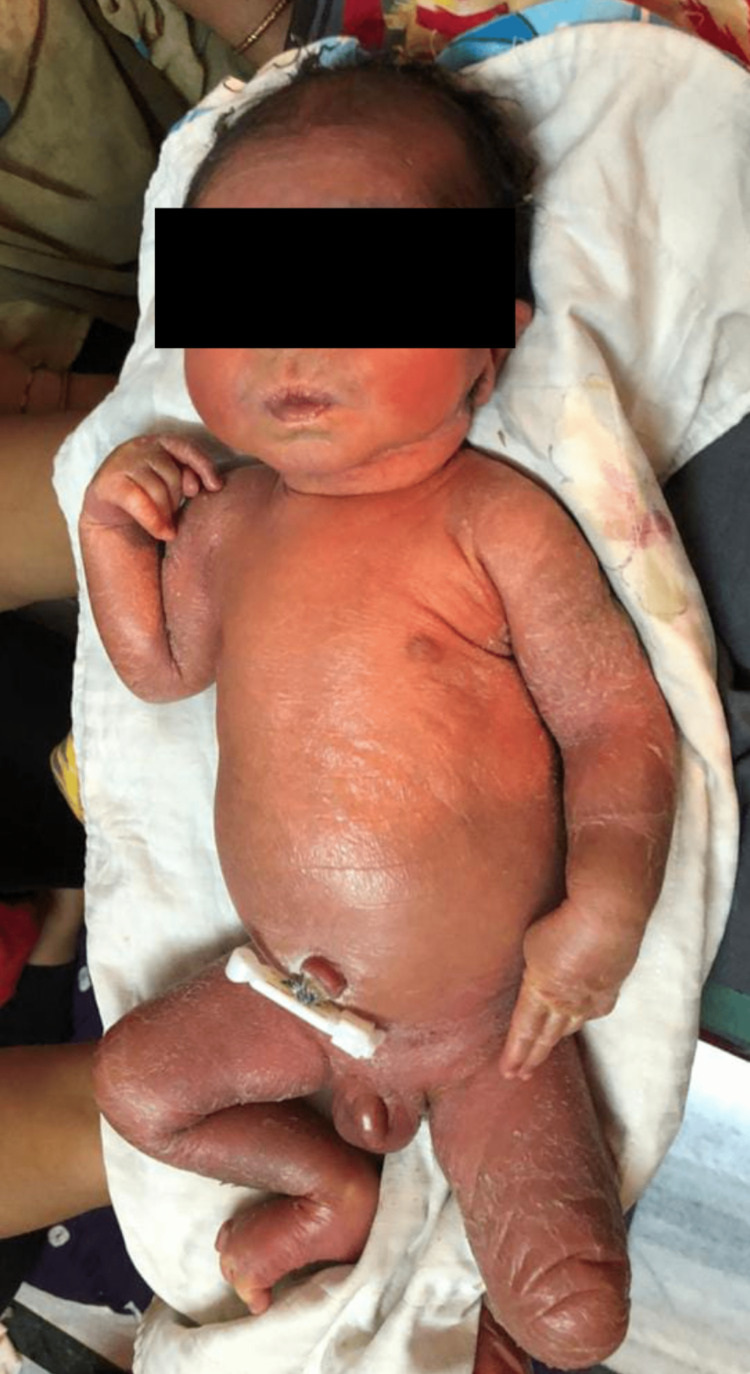
Patient’s condition at the time of birth. Skin was light brown in color, and there was exfoliation of the skin all over his body.

On examination, the patient’s temperature was normal, his pulse was approximately 120 beats per minute, his respiration rate was 25 breaths per minute, his blood pressure was approximately 80/50 mmHg, his weight was 8.4 kg, and his height was 68 cm. The patient was anemic, and abdominal distension was present. As seen in Figure 1, the patient’s skin was light brown in color, and there was exfoliation of the skin all over his body.

**Figure 2:**
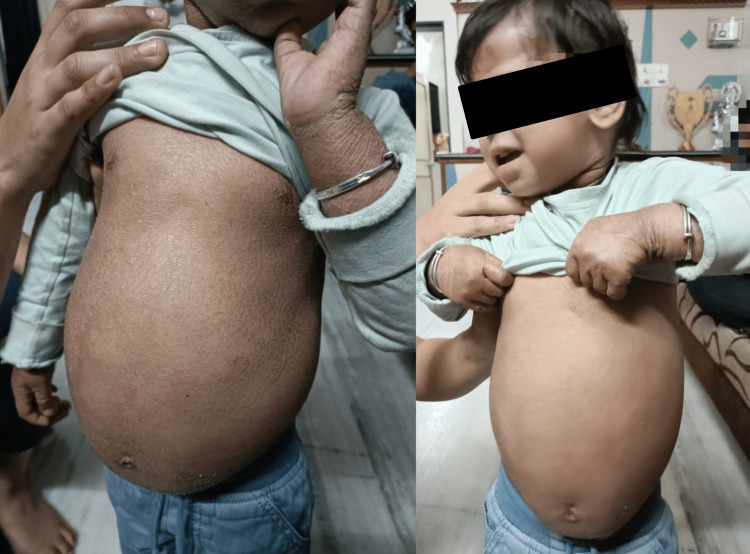
Patient’s condition at one year of age. Exfoliation of the skin with abdominal enlargement.

On physical examination, we observed redness of the skin; we also observed shiny skin over the groin and a whitish patch over both eyebrows. During a systemic examination of the abdomen, the liver was palpable at 10 cm, whereas the spleen was not palpable; no ascites were present, as seen in Figure 2.

We performed numerous tests on the patient. Ultrasonography revealed extensive hepatomegaly, but the patient’s complete blood count was normal. Urine protein excretion was elevated to 17.1 mg/dL (normal=12 mg/dL), whereas his fasting glucose level was normal. At first, he was diagnosed with a glycogen storage disease. To confirm, a liver tissue biopsy was done for this gross microscopic examination one core of pale, whitish soft liver tissue (1.5 cm) was taken. Microscopic examination showed that the liver tissue had diffuse, marked steatosis (predominantly macrovesicular); portal, extensive, frequent periportal fibrosis; occasional thin bridging fibrosis; and only minimal portal and mild lobular inflammation. Early-developing cirrhosis was considered. The features were suggestive of metabolic disorders and required further evaluation with relevant clinical and pathological tests. No malignancy was found.

Genetic testing of the patient’s mother showed heterozygous status for the c.683T>G (p.Leu229Ter) variant and wild-type (negative) status for the c.752A>C (p.His251Pro) variant in the ABHD5 gene (Table 1).

**Table 1:** Exome sequencing of the patient’s mother.

Exome sequencing test	
Gene and Transcript	Zygosity
ABHD5: c.683T>G (p.Leu229Ter)	Detected (Heterozygous)
ABHD5: c.752A>C (p.His251Pro)	Not detected

Genetic testing of the patient’s father showed wild-type (negative) status for the c.683T>G (p.Leu229Ter) variant and heterozygous status for the c.752A>C (p.His251Pro) variant in the ABHD5 gene (Table 2).

**Table 2:** Exome sequencing of the patient’s father.

Exome sequencing test	
Gene and transcript	Zygosity
ABHD5: c.683T>G (p.Leu229Ter)	Not detected
ABHD5: c.752A>C (p.His251Pro)	Detected (heterozygous)

Genetic testing of the patient revealed probable compound heterozygosity which was likely pathogenic and variant of uncertain significance (vus) variants consistent with the phenotype (Table 3).

**Table 3:** Summary of findings.

Gene and transcript	Location	Variant	Zygosity	Classification	Disease	Inheritance
ABHD5 (NM_016006.4)	Exon 5	c.683T>G (p.Leu229Ter)	Heterozygous	Likely pathogenic	Chanarin-Dorfman syndrome	Autosomal recessive
ABHD5 (NM_016006.4)	Exon 5	c.752A>C (p.His251Pro)	Heterozygous	Uncertain significance	Chanarin-Dorfman syndrome	Autosomal recessive

An amino acid quantitative urine analysis by tandem mass spectrometry (TMS) of the patient’s early-morning urine showed increased levels of aspartic acid, methionine, ethanolamine, isoleucine, and arginine. Serum alpha-fetoprotein level was high at 7.11 IU/mL (normal = 0.50-5.50 IU/mL). An amino acid quantitative analysis of LC-MS/MS EDTA-plasma showed decreased levels of alanine and leucine, and slightly increased levels of citrulline, glycine, and proline. We also performed a peripheral smear examination (Table 4).

**Table 4:** Results of peripheral smear examination.

Peripheral smear examination	
RBCS	Hypochromasia + Microcytosis + Anisocytosis
WBCs	Vacuoles seen in the cytoplasm of the leukocytes
Platelets	Adequate on peripheral smear
Hemoparasites	No malarial or filarial parasite seen
Abnormal cells	Granulocytes showed vacuolated cytoplasm
Impression	Mild anemia with the presence of vacuolations in leukocytes consistent with peripheral smear findings in Chanarin-Dorfman syndrome

Both parents’ lipid profiles were normal, as was the infant’s serum zinc level 63.34 µg/dL (normal = 64-118 µg/dL). Alanine transaminase and aspartate transaminase were elevated in the patient at approximately 65 U/L (normal = 7-56 U/L) and 60 U/L (normal = 8-45 U/L), respectively. 

The diagnosis of CDS was confirmed by exon sequencing, and the patient was prescribed multivitamin and multimineral supplements, vitamin D3, vitamin K, omega-3 fatty acid, vitamin E, and a low-fat diet. In addition, we advised his parents to combine skim-milk powder, medium chain triglycerides (MCTs) oil, and simyl MCT powder in 30 mL (i.e., one to two teaspoons), and give him the mixture every 60-90 min. Furthermore, we advised his parents to reduce the frequency of breastfeeding and increase his food intake, avoiding cow or bovine milk, sugar, baked goods, fried foods, oil, and ghee. As part of the treatment plan, we prescribed simyl or coconut oil (three tablespoons per day), and half a tablespoon of MCT oil in each meal. As a result, he showed improvement overall with no traces of lipid inclusions noted after treatment, and his lipid profile and liver enzyme levels returned to normal. The peripheral smear examination and contrast tomography of the abdomen revealed the absence of lipid-containing vacuoles.

## Discussion

Chanarin-Dorfman syndrome is a very rare autosomal recessive syndrome developing due to ABHD5/CGI58 gene mutation and is more prevalent in Mediterranean and Middle Eastern countries. In CDS, lipid accumulation occurs in various tissues as a result of abnormal catabolism of triacylglycerols. Normally, CGI58 proteins found on the surface of cytoplasmic lipid droplets activate lipase, leading to lipolysis. Mutations in the ABHD5/CGI58 gene prevent lipolysis, consequently leading to lipid accumulation in leukocytes, fibroblasts, liver, and muscle cells. Clinically, the disease presents with ichthyosis, hearing loss, hepatomegaly, splenomegaly, cirrhosis, cataract, myopathy, and mental retardation. Skin findings include dryness, erythema, hyperkeratosis, and ichthyosis [1]. The most common extracutaneous manifestation is hepatomegaly (60% of patients), followed by myopathy (59%), ocular manifestation (bilateral ectropion in 29% and cataract in 22%), neurosensory deafness (17%), and splenomegaly (13%) [3].

Our case presented with dry scaly skin with exfoliation all over the skin, redness over the majority of the skin, shiny skin over the groin area, and whitish patches over both eyebrows -- all of which are marked features of NLSD. In addition, some extracutaneous features (e.g., abdominal bloating and hepatomegaly) were present, and ophthalmic investigations revealed the sundrop sign and cataract during infancy. Because of allelic heterogeneity, the clinical presentation of CDS varies based on the type of underlying mutation present. To date, a total of 78 mutations related to CDS have been identified, including splice-site mutations, insertions, deletions, and nonsense and missense mutations, with the most frequently observed being c.594insC p.N209X [3].

Our case included a genetic study of both parents, in which both were revealed to have heterozygous variants on the ABHD5 gene on exon 5. The ABHD5(chr3:43756460);C.683T > G(p.Leu229Ter) variant was found in the mother’s sample, which is a stop-gained p.L228* variant in ABHD5 (NM016006.6) that has not been reported previously as either pathogenic or benign. The p.L228* variant is novel (i.e., not in any individuals) in gnomAD exomes and 1000 genomes. This ABHD5 gene variant is predicted to cause loss of normal protein function through protein truncation. Therefore, the p.L228* variant has been classified as “likely pathogenic.” The ABHD5 (Chr3:43756529);c.752A>C (p.His251Pro) variant was found in the father’s sample, which is a missense p.H251P variant in ABHD5 (NM_016006.6) that has not been reported previously as either pathogenic or benign. The p.H251P variant is observed in 1 out of 30,602 (0.0033%) alleles from individuals of a South Asian background in gnomAD exomes and in 1 out of 978 (0.1022%) alleles from individuals of a South Asian background in 1000 genomes. The p.H251P missense variant is predicted to be damaging by both SIFT and PolyPhen2. The histidine residue at codon 251 of ABHD5 is conserved in all mammalian species. The nucleotide c.752 in ABHD5 is predicted to be conserved by GERP++ and PhyloP across 100 vertebrates. Therefore, this variant has been classified as “uncertain significance.” A similar novel case with different variants has also been reported [10].

The most common and characteristic laboratory finding of CDS is May-Grünwald-Giemsa-negative lipid droplets (Jordans’ anomaly) in otherwise normal peripheral blood leukocytes. Before any clinical suspicion of CDS, a blood smear should be performed to screen for lipid inclusions in neutrophils [11]. The liver is reported to be the most frequently affected organ in CDS, our patient also in line with the common presentation presented with hepatomegaly and elevated liver enzymes and fatty liver. Severe fatty degeneration is a common histopathological finding in CDS. Although not frequently reported, splenomegaly is also possible [12].

Our case showed lipid inclusions on the peripheral smear. Sonography showed gross hepatomegaly with altered echogenicity and regular surface margin; mild splenomegaly was also present. Both kidneys showed cortical echogenicity with partial loss of corticomedullary differentiation suggesting medical renal disease. A biochemical test revealed elevated levels of alpha fetoprotein (7.11 IU/mL). A liver function test revealed elevated levels of direct bilirubin, high prothrombin time, and low hemoglobin. The patient also had elevated levels in his lipid profile, with increased LDL/HLD ratio and triglyceride levels. We suggested diet modification as a treatment option, as a diet low in fatty acids in addition to medium-chain triglyceride supplements may decrease hepatomegaly and normalize hepatic enzymes, especially when initiated early and in combination with vitamin E (10 mg/kg/day) and ursodeoxycholic acid (15-20 mg/kg/day). Another case study where a patient with NLSD with ichthyosis who was on this diet had a 50% decrease in liver size by the end of the first year of diet modification, and a notable improvement of skin lesions after being on this diet for five years. Vitamin E has been prescribed for the management of steatosis [13].

After we made our final diagnosis, our patient was started on a special diet that lacked long-chain fatty acids and was rich in medium-chain fatty acids. He was started on omega-3 fatty acid supplements, vitamin E, vitamin K, a multivitamin B complex, and minerals. As a result, he showed improvement overall with no traces of lipid inclusions noted after treatment, and his lipid profile and liver enzyme levels returned to normal.

## Conclusions

This case report highlights the management of a patient with CDS. This is a rare condition characterized by a wide spectrum of clinical manifestations. The presenting symptoms of skin peeling, anemia, hepatomegaly, neonatal jaundice, cataract, and elevated triglycerides, as well as the biopsy findings of early developing cirrhosis and macrovesicular steatosis, were consistent with the diagnosis. The identification of heterozygous alleles in both parents through genetic testing confirmed the hereditary nature of the syndrome. The treatment approach consisted of a comprehensive regimen that included multivitamins and multimineral supplements (e.g., vitamin D3, vitamin K, vitamin E, and omega-3 fatty acids), and the inclusion of medium-chain triglycerides in the patient’s diet. Following one year of strict adherence to a low-lipid diet, significant improvements were observed in both laboratory findings and clinical symptoms. Notably, there were no lipid-containing vacuoles in leukocytes, and the biopsy results returned to normal.

This case report underscores the importance of a multidisciplinary approach in managing rare conditions such as CDS. The successful outcome in this case highlights the potential benefits of targeted dietary modifications and the use of specific supplements in improving the clinical course of the disease. Further research and larger studies are required to establish standardized management protocols for patients with CDS.
